# [Corrigendum] Podoplanin-mediated TGF-β-induced epithelial-mesenchymal transition and its correlation with bHLH transcription factor DEC in TE-11 cells

**DOI:** 10.3892/ijo.2026.5854

**Published:** 2026-02-11

**Authors:** Yunyan Wu, Qiang Liu, Xu Yan, Yukio Kato, Makiko Tanaka, Sadaki Inokuchi, Tadashi Yoshizawa, Satoko Morohashi, Hiroshi Kijima

Int J Oncol 48: 2310-2320, 2016; DOI: 10.3892/ijo.2016.3445

Following the publication of the above paper, it was drawn to the Editor's attention by a concerned reader that, for the scratch-wound assay experiments shown in Fig. 8 on p. 2318, the 'control-siRNA/24 h' and 'podoplanin-siRNA/48 h' panels contained an overlapping section of data; such that these data, which were intended to show the results from differently performed experiments, appeared to have been derived from the same original source. Upon analyzing the data independently in the Editorial Office, it came to light that, in addition to control blots, the podoplanin blots were duplicated in Fig. 2A and B, and also in Fig. 3A and B, although it wasn't clear whether this was simply the way in which the authors had chosen to arrange the data in these figures, as the reported experimental conditions were the same in the respective figure parts (note that an Expression of Concern statement was also published for this paper: doi.org/10.3892/ijo.2025.5805).

Upon contacting the authors, they confirmed that the data had been included in Figs. 2 and 3 as intended, although they realized that an error had been made during the assembly of the scratch-wound assay images shown in Fig. 8. However, the authors had retained their original data, and the data for the podoplanin-siRNA/48 h' panel was shown incorrectly in this Figure. In the first instance, the authors wish to propose the following wording for the figure legend for Fig. 3, to clarify that the same podoplanin and β-actin bands were intended to have been shown in Figs. 2 and 3 (the added text is highlighted in bold): 'Figure 3. The protein expression of podoplanin and EMT-related markers was regulated by TGF-β. TE-11 cells were treated as indicated in Fig. 2. These cells were then lysed, and the lysates were subjected to western blot analyses of pSmad2, Smad2/3, Slug, podoplanin, vimentin, N-cadherin, E-cadherin, Claudin-4, DEC1, DEC2, and β-actin. **The data of podoplanin and β-actin used in (A) were shown again in (B) as necessary**. Two representative results of at least four independent experiments with similar outcomes are shown'.

Textual corrections should also be noted in this Corrigendum: First, in the figure legend for Fig. 8, the P-value for significance should have been written as '^*^P<0.05', not as '^*^P<0.001'. Secondly, in the Results section, the '*Podoplanin was closely involved in the invasion and migration in TE-11 cells*' subsection on p. 2314, sentences 5−7 in this paragraph should have been written as follows ('at 24 h' should have been included in the sixth sentence): 'In order to evaluate the ability of migration, wound-healing assay was introduced in podoplanin siRNA-transfected cells. Remaining wound length was measured after 0, 24 and 48 h of podoplanin siRNA transfection, and a significant difference between the control siRNA-transfected group and the podoplanin siRNA-transfected group **at 24 h** was attained (Fig. 8). Morphologically, a small amount of the TE-11 cells in the middle of the chamber was dead in the podoplanin-siRNA transfected group, especially at the 24-h time-point'.

The revised version of Fig. 8, now showing the correct data for the 'podoplanin-siRNA/48 h' experiment, is shown on the next page. The authors confirm that the error made in assembling Fig. 8 did not have a major impact on the conclusions reported in the above article, and they thank the Editor of *International Journal of Oncology* for allowing them the opportunity to publish a Corrigendum. Furthermore, all the authors agree to the publication of this Corrigendum, and apologize to the readers for any inconvenience caused.

## Figures and Tables

**Figure 8:**
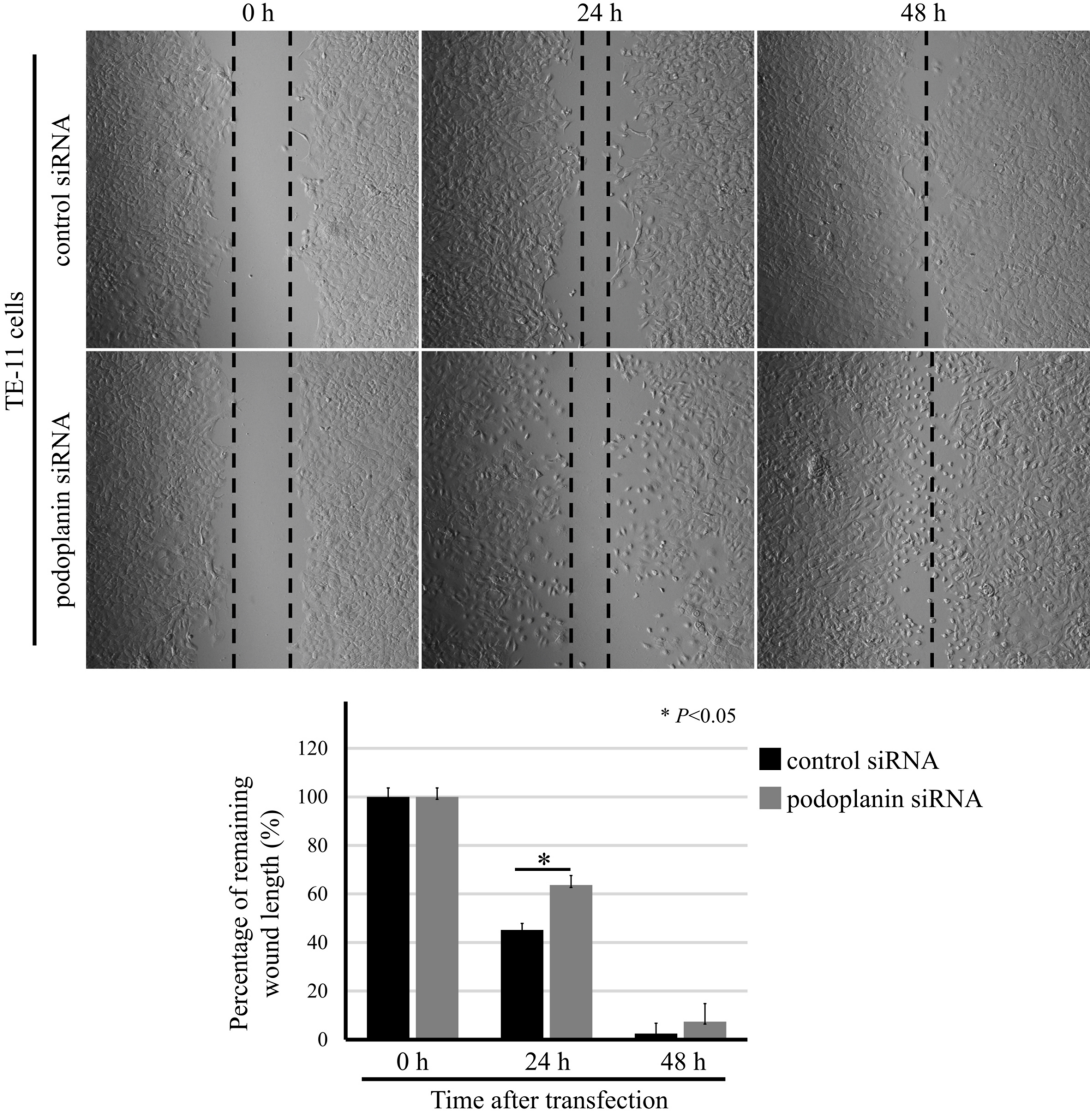
Podoplanin siRNA delayed the speed of wound-healing in TE-11 cells. TE-11 cells were transfected with control siRNA or siRNA against podoplanin for 24 h, a wound was made with a pipette tip and photographs of the wounded area were taken periodically. The remaining wound length was measured after 0, 24 and 48 h. The top panel shows representative photographs, and the bottom panel shows the quantitative data. Each value represents the mean ± SEM (bars) of three independent experiments (^*^P<0.05, compared with the control siRNA, according to the Student's t-test).

